# Fear and Medical Misinformation Regarding Risk of Progression or Recurrence Among Patients with Breast Cancer

**DOI:** 10.1001/jamanetworkopen.2025.49809

**Published:** 2025-12-29

**Authors:** David Gibbes Miller, Kaitlyn Lapen, Edward Christopher Dee, Sam Meske, Marisa C. Weiss, Junzo P. Chino, Fumiko Chino

**Affiliations:** 1 Memorial Sloan Kettering Cancer Center, New York, New York; 2 Breastcancer.org, Ardmore, Pennsylvania; 3 Lankenau Institute for Medical Research, Wynnewood, Pennsylvania; 4 Duke Cancer Institute, Durham, North Carolina; 5 MD Anderson Cancer Center, Houston, Texas

## Abstract

**Importance:**

Misinformation about cancer treatment is increasingly common. It is not known whether exposure to such misinformation is related to fear of cancer recurrence or adherence to evidence-based treatment.

**Objective:**

To evaluate exposure to misinformation among adults with a history of breast cancer and assess its association with recurrence fears and treatment adherence.

**Design, Settings, and Participants:**

In this survey study, a patient-facing online survey was administered by Breastcancer.org from July to August 2023. Eligible participants were adult US residents who received a breast cancer diagnosis within the past 10 years. Surveys assessed patient exposure to non–evidence-based factors purported to affect cancer progression or recurrence risk (ie, misinformation).

**Main Outcomes and Measures:**

Survey answers on misinformation. Secondary outcomes included recurrence fears, which were evaluated through the validated Fear of Cancer Recurrence Inventory-Short Form (scored on a 0-36 scale, with scores of 22 or higher indicating clinical relevance); χ^2^ tests assessed the association between misinformation exposure and both fear of recurrence and treatment adherence.

**Results:**

A total of 997 patients completed the survey (median [IQR] age, 62 [53-69] years; 48 Black [5%], 43 Hispanic [4%], 853 White [86%]; 748 married [75%] and 765 with college education [78%]). Five hundred twenty patients (52%) were undergoing active cancer treatment. A total of 761 participants (76%) reported encountering misinformation about breast cancer; 651 (65%) reported misinformation exposure about factors that increase the risk of cancer progression or recurrence (eg, sugar, deodorant, cell phones, vaccines). Misinformation exposure about factors that decrease risk (eg, organic food, vitamins or supplements, and alkaline diets) were reported by 542 (54%). The median (IQR) fear of cancer recurrence score was 19 (13-24), with 262 participants (38%) reporting clinically significant fear. Self-reported treatment adherence was 76% overall (758 participants). Misinformation exposure was not associated with clinically significant fear of recurrence (38% vs 35%; χ^2^ = 0.53; *P* = .47) or treatment nonadherence (23% vs 26%; χ^2^ = 0.90; *P* = .34). On multivariable analysis of sociodemographic features, patients of Hispanic ethnicity had higher odds of misinformation exposure (adjusted odds ratio, 2.96; 95% CI, 1.15-10.05; *P* = .04), although this association did not persist on multivariable analysis including fear of recurrence and treatment adherence.

**Conclusions:**

In this survey study of patients with breast cancer, exposure to medical misinformation was common, underscoring the need for better survivorship communication with patients; fear of recurrence was not associated with exposure to misinformation. Further research on how patients process medical misinformation is essential, especially in populations at highest risk for misinformation spread.

## Key Points

**Question:**

Among patients with breast cancer, how common is exposure to medical misinformation, and is such exposure associated with fear of cancer recurrence or lower adherence to treatment?

**Findings:**

In this survey study of 997 individuals with a history of breast cancer, 76% reported that they encountered medical misinformation. Exposure to misinformation was not significantly associated with clinically significant fear of cancer recurrence or self-reported treatment nonadherence.

**Meaning:**

In this study, exposure to medical misinformation was not associated with fear of cancer recurrence or treatment nonadherence, but exposure to such misinformation was reported by a vast majority of participants.

## Teaser

This survey study of US adults with a history of breast cancer reports the prevalence of misinformation and its association with recurrence fears and treatment adherence.

## Introduction

Medical misinformation is information that is inaccurate or misleading according to the best available evidence.^1^ Misinformation is a growing concern in the US, with rapid proliferation through online platforms and social media.^2,3,4,5,6^ A 2023 survey^7^ of US patients with cancer showed that the vast majority had been exposed to cancer treatment misinformation on social media. Most of these participants thought at least some of the misinformation was true and were willing to share the misinformation with others. There is concern that exposure to misinformation may be associated with negative health behaviors,^8,9^ poor medical decision-making processes,^10^ mistrust in clinicians, refusal of evidence-based treatments, or pursuit of expensive and/or dangerous alternative treatments.^11,12,13^ A 2025 study^14^ has also shown that patients worry about seemingly contradictory advice given by experts, and only about half of cancer survivors trust cancer information from scientists. However, the extent to which exposure to cancer misinformation may impact behaviors such as treatment adherence is poorly understood.

Fear of cancer recurrence refers to worry or concern about cancer returning or progressing^15^; it is common among patients with breast cancer and often persists after treatment completion.^16,17,18^ Recurrence fears can harm quality of life, psychological well-being, and health care utilization.^18,19,20^ Some research shows associations between exposure to misinformation and health-related anxiety or behavior change. For example, during the COVID-19 pandemic, there was evidence that individuals who shared misinformation experienced approximately 2 times the level of anxiety when compared with individuals who did not share misinformation.^21^ Other researchers point out that misinformation, which may meet individuals’ unmet psychological needs, is predominantly linked to harmful social and health consequences such as vaccine refusal.^6,22^ Within oncology specifically, there is evidence that fear of cancer recurrence is associated with increased complementary and alternative medicine use.^19,23^ However, it is not known whether fear of cancer recurrence is associated with exposure to medical misinformation.

This study sought to evaluate breast cancer survivors’ exposure to misinformation about cancer recurrence or progression, and assess its association with recurrence fears and treatment adherence. We hypothesized that participants who have been exposed to cancer misinformation may report higher fears of cancer recurrence and lower treatment adherence.

## Methods

### Survey Design

An anonymous, cross-sectional online survey was administered between July and August 2023 by Breastcancer.org, with online, social media, and email recruitment. Digital ads were shown on Breastcancer.org in addition to recruitment posts on Instagram, LinkedIn, Threads, and Facebook; emails were sent to the Breastcancer.org mailing list. Eligible participants were US residents, aged 18 years or older, who received breast cancer diagnoses within the past 10 years. Surveys were available in English or Spanish. Participants provided written informed consent electronically prior to completion. The Solutions institutional review board reviewed this study and found it to be exempt. This study adhered to the American Association for Public Opinion Research (AAPOR) best practice guidelines for survey studies.

The survey was designed by clinicians, researchers, and patient advocates, with patient testing for face validity prior to launch. Participants were presented with a list of factors commonly claimed to influence cancer recurrence or progression. They were asked whether they had encountered information that certain factors could increase (eg, sugar consumption, deodorant use, and vaccines) or decrease (eg, organic food, vitamins or supplements, and cleanses) cancer recurrence or progression risk. Three co-investigators (M.W., J.C., and F.C.) reached consensus on factors considered misinformation (ie, no evidence-based association with breast cancer). Participants who reported encountering these factors were considered to have been exposed to medical misinformation.

Fear of recurrence, defined as fear that cancer could return or progress in the same place or in another part of the body, was assessed using the 9-question validated Fear of Cancer Recurrence Inventory-Short Form (FCRI-SF). Scores ranged from 0 to 36, with higher scores indicating greater fear; scores of 22 or higher are considered clinically significant as per previously published literature.^15,24^ Demographic and treatment adherence information was self-reported by participants. Categories for race and ethnicity were Asian, Hispanic or Latino, non-Hispanic Black, non-Hispanic White, and other (which included multiracial, Middle Eastern or North African, West Indian Barbados or French, Native American or American Indian, and prefer not to answer). Race and ethnicity have previously been found to be associated with exposure to medical misinformation and were therefore included as variables of interest. Participants were asked if they stopped treatment early or accepted less treatment than prescribed; any yes responses were considered suboptimal adherence.

### Statistical Analysis

Descriptive statistics summarized self-reported participant demographics and survey responses. χ^2^ Tests assessed the association between misinformation exposure and significant recurrence fear (FCRI-SF score of 22 or higher), and between misinformation exposure and treatment adherence. Given that some patients had metastatic disease and may fear progression rather than recurrence per se, a sensitivity analysis with χ^2^ tests excluding participants with metastatic cancer was also performed. Another sensitivity analysis was conducted using the Wilcoxon rank-sum test to compare continuous FCRI-SF composite scores between participants with and without misinformation exposure, recognizing that potential differences in fear of recurrence may not be reflected by the threshold score of 22 used to define clinically meaningful fear of recurrence.

A multivariable logistic regression explored associations between sociodemographic factors and exposure to misinformation. The model was constructed using a conceptually driven approach, with sociodemographic variables selected a priori based on their theoretical relevance to misinformation exposure. Categories with very small sample sizes were excluded to ensure model stability. Missing data were handled using complete-case analysis for the multivariable logistic regression; participants with missing values were excluded prior to multivariable analysis. A separate multivariable model also included sociodemographic variables, clinically significant recurrence fear, and treatment adherence.

Statistical significance was defined as a 2-sided *P* value <.05. Statistical analyses were conducted using RStudio version 4.3.3 (R Foundation for Statistical Computing).

## Results

Overall, 997 patients with breast cancer were eligible and answered some survey questions (Table 1). Of these, 988 answered all misinformation questions (99% completion rate); those who did not answer the misinformation items were considered not to have been exposed misinformation in the analysis. Median (IQR) participant age was 62 (53-69) years; the majority were married (748 [75%]) and had completed at least a college degree (765 [78%]). Overall, there were 43 (4%) Hispanic respondents, 48 (5%) non-Hispanic Black respondents, and 853 (86%) non-Hispanic White respondents. At the time of survey completion, 520 (52%) were undergoing active cancer treatment and 65 (7%) had metastatic disease.

**Table 1.** Patient Characteristics

Variable	Patients, No. (%)			*P* value
	Overall (N = 997)	Not exposed to misinformation (n = 236)	Exposed to misinformation (n = 761)	
Age, median (IQR), y	62 (53-69)	63 (54-70)	62 (52-69)	.07^a^
Race and ethnicity				
Asian	26 (3)	6 (3)	20 (3)	.24^b^
Hispanic or Latino	43 (4)	4 (2)	39 (5)	
Non-Hispanic Black	48 (5)	10 (4)	38 (5)	
Non-Hispanic White	853 (86)	209 (89)	644 (85)	
Other or unknown^c^	27 (3)	7 (3)	20 (3)	
Highest level of education				
High school or less	58 (6)	14 (6)	44 (6)	.71^b^
Some college	167 (17)	36 (15)	131 (17)	
Associate or bachelors	431 (44)	110 (47)	321 (43)	
Advanced degree	334 (34)	76 (32)	258 (34)	
Marital status				
Married or partnered	748 (75)	178 (75)	570 (76)	.91^b^
Single	87 (9)	23 (10)	64 (9)	
Divorced or separated	104 (10)	23 (10)	81 (11)	
Widowed	52 (5)	12 (5)	40 (5)	
Employment status				
Employed	475 (48)	104 (44)	371 (49)	.42^d^
Retired	397 (40)	108 (46)	289 (38)	
Homemaker	33 (3)	7 (3)	26 (4)	
Student	2 (<1)	1 (<1)	1 (<1)	
On leave or disability	47 (5)	8 (3)	39 (5)	
Unemployed or not looking for a job	35 (4)	8 (3)	27 (4)	
Insurance status				
Private insurance	562 (56)	130 (55)	432 (57)	.27^d^
Medicare	375 (38)	97 (41)	278 (37)	
Medicaid	34 (3)	8 (3)	26 (3)	
No insurance	1 (<1)	0	1 (<1)	
Other^e^	24 (2)	1 (<1)	23 (3)	
Breast cancer status				
Early-stage	582 (59)	141 (60)	441 (58)	.87^b^
Locally advanced	347 (35)	79 (34)	268 (35)	
Metastatic	65 (7)	15 (6)	50 (7)	
Treatment status				
On active treatment	520 (52)	127 (54)	393 (52)	.64^b^
Treatment naive	24 (2)	7 (3)	17 (2)	
Completed treatment	453 (45)	102 (43)	351 (46)	

^a^ Wilcoxon rank-sum test.

^b^ Pearson χ^2^ test.

^c^ Other or unknown race and ethnicity includes the following responses: multiracial; Middle Eastern or North African; West Indian Barbados or French; Native American or American Indian; and prefer not to answer.

^d^ Fisher exact test.

^e^ Other insurance status includes faith-based insurance groups, coverage only from health savings accounts, Kaiser plans, and military insurance programs.

Most participants (761 [76%]) reported encountering misinformation about factors purported to influence cancer progression or recurrence risk. Overall, 651 (65%) reported hearing about factors purported to increase the risk of progression or recurrence—the most frequent factor cited was that sugar increases this risk (609 [61%]). Participants also reported hearing that deodorant (224 [22%]), vaccines (78 [8%]), cell phones (78 [8%]), and bra type (75 [8%]) increased risk. Over half of patients (542 [54%]) reported hearing misinformation regarding factors believed to decrease risk of progression or recurrence—eating organic food was cited as the most frequent factor that decreases risk (409 [41%]). Participants also reported hearing that taking oral vitamins or supplements (285 [29%]), consuming an alkaline diet or alkalized water (121 [12%]), receiving vitamin infusions (65 [7%]), using cleanses (51 [5%]), using essential oils (52 [5%]), and using oxygen therapy (42 [4%]) decreased risk (Figure).

**Figure.**
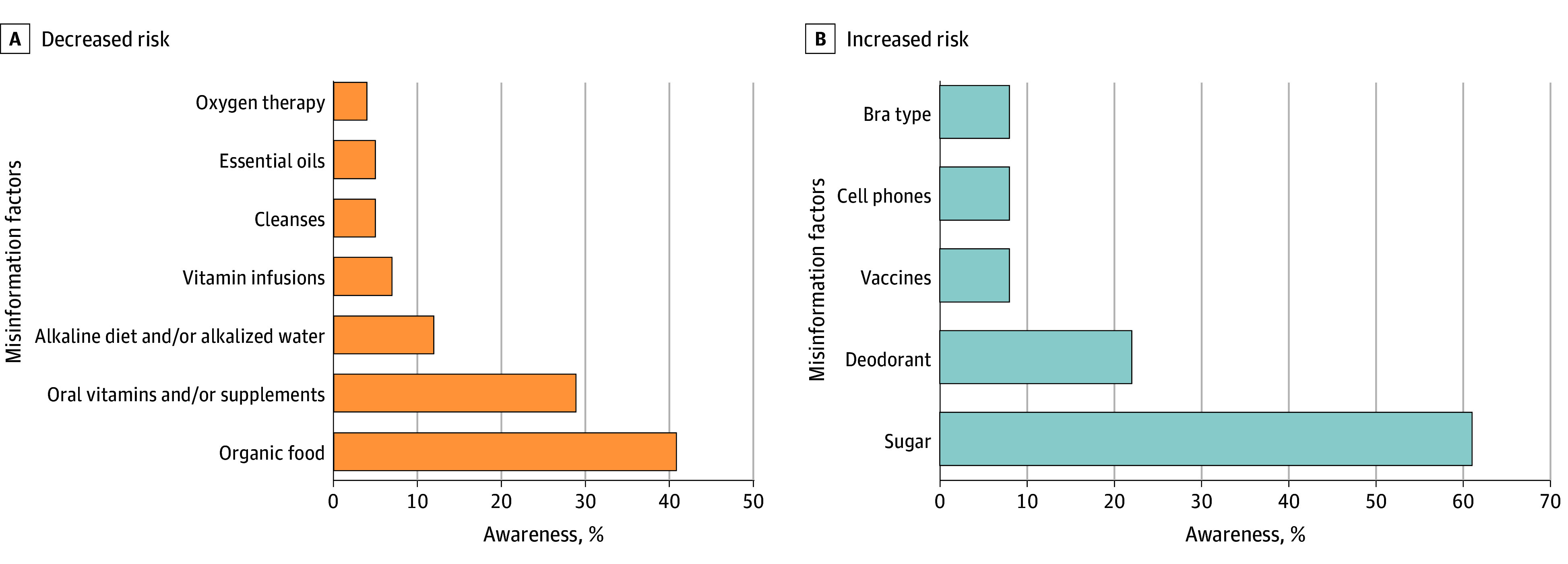
Awareness of Misinformation Factors That May Decrease or Increase Risk of Cancer Progression or Recurrence

Fear of recurrence scores (FCRI-SF) were available for 695 participants (70%). The median (IQR) FCRI-SF score was 19 (13-24), with 262 (38%) reporting clinically significant fear (ie, scoring 22 or higher). Misinformation exposure was not associated with clinically significant fear of cancer recurrence (rate of clinically significant fear: 38% of patients exposed to misinformation vs 35% of those not exposed; χ^2^ = 0.53; *P* = .47). Self-reported treatment adherence to all recommended therapies was 76%. There was also no association between exposure to misinformation and treatment nonadherence (nonadherence rate: 23% of patients exposed to misinformation vs 26% of those not exposed; χ^2^ = 0.90; *P* = .34). On a sensitivity analysis excluding participants with metastatic disease, misinformation exposure again was not associated with either clinically significant fear of cancer recurrence (41% vs 38%; χ^2^ = 0.62; *P* = .43) or treatment adherence (22% vs 25%; χ^2^ = 0.82; *P* = .36). In an additional sensitivity analysis treating FCRI-SF scores as continuous, participants exposed to misinformation had slightly higher FCRI-SF scores compared with those not exposed (median [IQR], 19 [13-25] vs 18 [12-23]); however, this difference was not statistically significant (Wilcoxon rank-sum test, *W* = 40 417; *P* = .10).

On multivariable analysis including only sociodemographic factors, participants of Hispanic ethnicity had higher odds of misinformation exposure than White participants (adjusted odds ratio, 2.96; 95% CI, 1.15-10.05; *P* = .04) (Table 2). However, when clinically meaningful fear of recurrence and treatment adherence data were added to the multivariable analysis, there were no variables with independent associations with misinformation exposure (eTable in Supplement 1).

**Table 2.** Multivariable Logistic Regression of Sociodemographic Features Associated with Awareness of Misinformation

Variable	Adjusted OR (95% CI)	*P* value
Age		
Per 10-y increase	0.87 (0.70-1.09)	.23
Race and ethnicity		
Non-Hispanic White	1 [Reference]	NA
Asian	0.96 (0.39-2.71)	.94
Hispanic or Latino	2.96 (1.15-10.05)	.04
Non-Hispanic Black	1.05 (0.51-2.36)	.89
Other or unknown	0.76 (0.32-2.01)	.55
Highest level of education		
Postgraduate degree	1 [Reference]	NA
Associate or bachelors	0.83 (0.59-1.18)	.30
Some college	1.14 (0.72-1.82)	.58
High school	0.89 (0.46-1.81)	.74
Marital status		
Married or partnered	1 [Reference]	NA
Single	0.76 (0.45-1.32)	.32
Divorced or separated	1.08 (0.65-1.84)	.78
Widowed	1.21 (0.62-2.54)	.59
Breast cancer status		
Early-stage	1 [Reference]	NA
Locally advanced	0.98 (0.71-1.36)	.89
Metastatic	1.16 (0.62-2.27)	.65
Treatment status		
On active treatment	1 [Reference]	NA
Treatment naïve	0.98 (0.38-2.81)	.96
Completed treatment	1.18 (0.86-1.62)	0.31
Employment status		
Employed	1 [Reference]	NA
Retired	0.75 (0.47-1.19)	.22
Homemaker	0.91 (0.38-2.42)	.84
Student	NR^a^	NR
On leave or disability	1.26 (0.57-3.07)	.59
Unemployed	0.96 (0.43-2.38)	.93
Insurance status		
Private insurance	1 [Reference]	NA
Medicare	1.25 (0.77-2.04)	.37
Medicaid	0.98 (0.42-2.49)	.96
No insurance	NR^a^	NR
Other	NR^a^	NR

Abbreviations: NA, not applicable; NR, not reported; OR, odds ratio.

^a^ Results that were not reported had too few participants.

## Discussion

In this national, online survey-based study, three-fourths of breast cancer survivors had heard misinformation about factors purported to influence cancer recurrence. Although one-third of the sample had clinically relevant fear of recurrence and a quarter had some form of nonadherence, these were not associated with misinformation. Given the rise of misinformation and its known detrimental effects on patient outcomes, it remains clear that strong survivorship education and communication is vital.^25,26^

This high rate of exposure to misinformation in our survey is comparable with other studies of medical misinformation among cancer patients. One study found that 71% of surveyed survivors were exposed to misinformation, with exposure significantly higher among those with cancer than those without.^7^ A 2018 survey done by the American Society of Clinical Oncology (ASCO) found that over a third of US residents (39%) believe cancer can be cured solely through alternative therapies such as oxygen therapy, diet, vitamins, and minerals.^27^ A 2019 ASCO follow-up survey found misconceptions about factors that may cause cancer including 16% who believe that cell phones cause cancer.^28^

Misinformation can proliferate through social media,^29^ food or dietary supplement packaging,^30^ or even from other health care professionals.^31^ Social media platforms, which patients with cancer may use as support networks, are particularly important sources of medical misinformation,^7,12^ underscoring the need for better communication and information sharing with oncology patients in nontraditional or online formats.^13,32^ There may be opportunities for both institutional actors and individual clinicians to improve trust in medicine and combat misinformation. For example, there is growing research on how to improve the “psychological immunity” of the public to misinformation, including efforts to promote true medical information that may spread within patient communities.^33^ Numerous other approaches have been suggested or evaluated, including creating intervention toolboxes^34^ or conversation guides^35^ for clinicians, automatically flagging false social media posts online,^36^ promoting narrative-based corrections to health misinformation in the media,^37^ and partnering with people affected by cancer to codesign educational resources that improve digital health literacy.^38^

We found no correlation between awareness of misinformation and cancer recurrence fears or treatment adherence. One possible explanation for this finding is that patients may not internalize all misinformation they encounter. Other factors such as preexisting health beliefs, relationships with health care professionals, psychosocial support, and health literacy may influence behavior or health beliefs more than misinformation alone. Alternatively, patients may act upon misinformation in a manner that does not cause anxiety or change their treatment. For example, two-thirds of patients in our study reported hearing that sugar consumption increases risk of cancer progression. Some of these individuals may have responded by adopting a low-sugar diet, although this behavior may not affect their treatment adherence. While our study did not find associations between misinformation exposure and recurrence fears or treatment adherence, further investigation is important to further characterize how health misinformation may impact patient behavior, well-being, and health care utilization.

On our multivariable analysis of sociodemographic features, participants with Hispanic ethnicity had higher odds of misinformation exposure than White participants. Although the association did not remain significant when additional variables were added to the analysis, this attenuation may reflect model overfitting or reduced statistical power rather than a true absence of effect. The exclusion of roughly 30% of participants who did not complete the fear of recurrence questions likely reduced power and contributed to the loss of significance. Other research has shown that Hispanic communities may be uniquely at risk for misinformation. For example, certain populations including Hispanic patients were shown to be more likely to accept non–evidence-based treatments during the COVID-19 pandemic.^39,40^ Tailored information campaigns that reflect community experiences may have the best outcomes. For example, one study showed that targeted social media posts from Black and Hispanic or Latinx influencers were successful at improving parent attitudes and intentions to vaccinate their children for HPV.^41^

### Limitations

There are several limitations to our study. This survey recruited from BreastCancer.org, and our sample may not reflect those with less access to or interest in online communities. Additionally, our survey assessed exposure to misinformation but did not assess information sources (eg, social media, friends, clinicians) or participant beliefs or intentions to act on misinformation, potentially limiting how these findings translate into interventions. There may also have been unmeasured confounding factors not captured in our analysis, including health literacy, digital information health-seeking behaviors, and baseline anxiety levels, that could be associated with exposure to misinformation. Additionally, FCRI-SF scores were available for only 70% of participants, potentially limiting our ability to identify associations between misinformation exposure and fear of recurrence. Finally, patients with metastatic breast cancer were included in the initial analysis of FCRI-SF scores, although patients with metastatic disease may fear cancer progression rather than recurrence. To account for this, we performed a sensitivity analysis excluding patients with metastatic disease, which also did not find significant associations between exposure to misinformation and fears of cancer recurrence.

## Conclusions

In this survey study of breast cancer survivors, the vast majority of participants reported exposure to medical misinformation, although this exposure was not associated with fear of cancer recurrence or treatment adherence. Further research is necessary to understand the impacts of medical misinformation on cancer survivors, and to develop strategies to improve health care communication and evidence-based knowledge about cancer.

## Notes

Supplement 1. **eTable. **Multivariable Logistic Regression of Variables Associated with Awareness of Misinformation

Supplement 2.
Data Sharing Statement

## References

[1] Vraga EK, Bode L. Defining misinformation and understanding its bounded nature: using expertise and evidence for describing misinformation. Polit Commun. 2020;37(1):136-144. doi:10.1080/10584609.2020.1716500

[2] Office of the Surgeon General. Confronting health misinformation: the US Surgeon General’s advisory on building a healthy information environment. US Department of Health and Human Services. Published online 2021. Accessed October 21, 2024. https://www.ncbi.nlm.nih.gov/books/NBK572169/34283416

[3] Afful-Dadzie E, Afful-Dadzie A, Egala SB. Social media in health communication: a literature review of information quality. Health Inf Manag. 2023;52(1):3-17. doi:10.1177/1833358321992683 33818176

[4] Suarez-Lledo V, Alvarez-Galvez J. Prevalence of health misinformation on social media: systematic review. J Med Internet Res. 2021;23(1):e17187. doi:10.2196/17187 33470931 PMC7857950

[5] Wang Y, McKee M, Torbica A, Stuckler D. Systematic literature review on the spread of health-related misinformation on social media. Soc Sci Med. 2019;240:112552. doi:10.1016/j.socscimed.2019.112552 31561111 PMC7117034

[6] Khullar D. Social media and medical misinformation: confronting new variants of an old problem. JAMA. 2022;328(14):1393-1394. doi:10.1001/jama.2022.17191 36149664

[7] Lazard AJ, Nicolla S, Vereen RN, . Exposure and reactions to cancer treatment misinformation and advice: survey study. JMIR Cancer. 2023;9:e43749. doi:10.2196/43749 37505790 PMC10422174

[8] Miller J, Prichard I, Hutchinson A, Wilson C. The relationship between exposure to alcohol-related content on Facebook and predictors of alcohol consumption among female emerging adults. Cyberpsychol Behav Soc Netw. 2014;17(12):735-741. doi:10.1089/cyber.2014.0337 25489875

[9] Vraga EK, Bode L, Tully M. The effects of a news literacy video and real-time corrections to video misinformation related to sunscreen and skin cancer. Health Commun. 2022;37(13):1622-1630. doi:10.1080/10410236.2021.1910165 33840310

[10] Shaverdian N, Kishan AU, Veruttipong D, . Impact of the primary information source used for decision making on treatment perceptions and regret in prostate cancer. Am J Clin Oncol. 2018;41(9):898-904. doi:10.1097/COC.0000000000000387 28537990

[11] Swire-Thompson B, Johnson S. Cancer: a model topic for misinformation researchers. Curr Opin Psychol. 2024;56:101775. doi:10.1016/j.copsyc.2023.101775 38101247 PMC10939853

[12] Johnson SB, Parsons M, Dorff T, . Cancer misinformation and harmful information on Facebook and other social media: a brief report. J Natl Cancer Inst. 2022;114(7):1036-1039. doi:10.1093/jnci/djab141 34291289 PMC9275772

[13] Giusti R, Daniele G, Loupakis F. The role of social media in fueling bias in oncology. JAMA Oncol. 2025;11(7):683-684. doi:10.1001/jamaoncol.2025.0617 40272830

[14] Godinich BM, Khanjani N, Fuller CD, Chino F. Cancer misinformation and trust in doctors and scientists among cancer survivors. J Clin Oncol. 2025;43(16)(suppl):11124-11124. doi:10.1200/JCO.2025.43.16_suppl.11124

[15] Smith AB, Costa D, Galica J, . Spotlight on the Fear of Cancer Recurrence Inventory (FCRI). Psychol Res Behav Manag. 2020;13:1257-1268. doi:10.2147/PRBM.S231577 33376421 PMC7762428

[16] Ellegaard MBB, Grau C, Zachariae R, Bonde Jensen A. Fear of cancer recurrence and unmet needs among breast cancer survivors in the first five years—a cross-sectional study. Acta Oncol. 2017;56(2):314-320. doi:10.1080/0284186X.2016.1268714 28093034

[17] Dunn LB, Langford DJ, Paul SM, . Trajectories of fear of recurrence in women with breast cancer. Support Care Cancer. 2015;23(7):2033-2043. doi:10.1007/s00520-014-2513-8 25524004 PMC5469210

[18] Mutsaers B, Jones G, Rutkowski N, . When fear of cancer recurrence becomes a clinical issue: a qualitative analysis of features associated with clinical fear of cancer recurrence. Support Care Cancer. 2016;24(10):4207-4218. doi:10.1007/s00520-016-3248-5 27169700

[19] Thewes B, Butow P, Bell ML, ; FCR Study Advisory Committee. Fear of cancer recurrence in young women with a history of early-stage breast cancer: a cross-sectional study of prevalence and association with health behaviours. Support Care Cancer. 2012;20(11):2651-2659. doi:10.1007/s00520-011-1371-x 22328003

[20] Zhang X, Sun D, Qin N, Liu M, Jiang N, Li X. Factors correlated with fear of cancer recurrence in cancer survivors: a meta-analysis. Cancer Nurs. 2022;45(5):406-415. doi:10.1097/NCC.0000000000001020 34560707

[21] Verma G, Bhardwaj A, Aledavood T, De Choudhury M, Kumar S. Examining the impact of sharing COVID-19 misinformation online on mental health. Sci Rep. 2022;12(1):8045. doi:10.1038/s41598-022-11488-y 35577820 PMC9109204

[22] Douglas KM, Uscinski JE, Sutton RM, . Understanding conspiracy theories. Polit Psychol. 2019;40(S1):3-35. doi:10.1111/pops.12568

[23] Burstein HJ, Gelber S, Guadagnoli E, Weeks JC. Use of alternative medicine by women with early-stage breast cancer. N Engl J Med. 1999;340(22):1733-1739. doi:10.1056/NEJM199906033402206 10352166

[24] Simard S, Savard J. Fear of Cancer Recurrence Inventory: development and initial validation of a multidimensional measure of fear of cancer recurrence. Support Care Cancer. 2009;17(3):241-251. doi:10.1007/s00520-008-0444-y 18414902

[25] Johnson PL. Confronting medical misinformation: leveraging our pandemic experiences. JAMA Intern Med. 2025;185(11):1307-1308. doi:10.1001/jamainternmed.2025.4072 40853556

[26] Rosenbaum ARP. A new age for trust in medicine. JAMA Oncol. 2025;11(10):1134-1135. doi:10.1001/jamaoncol.2025.2876 40875245

[27] Lee J. National cancer opinion survey reveals American attitudes toward alternative medicine, marijuana use. ASCO Daily News. Published online December 20, 2018. Accessed October 23, 2025. https://dailynews.ascopubs.org/do/10.5555/adn.18.190015/full/

[28] American Society of Clinical Oncology. National ASCO survey finds major gaps in Americans’ knowledge of cancer prevention, e-cigarettes, and end-of-life care. ASCO website. Published online October 29, 2019. Accessed October 23, 2025. https://www.asco.org/news-initiatives/policy-news-analysis/national-asco-survey-finds-major-gaps-americans-knowledge

[29] Nickel B, Moynihan R, Gram EG, . Social media posts about medical tests with potential for overdiagnosis. JAMA Netw Open. 2025;8(2):e2461940. doi:10.1001/jamanetworkopen.2024.61940 40009378 PMC11866028

[30] Assadourian JN, Peterson ED, Navar AM. Label statements and perceived health benefits of dietary supplements. JAMA Netw Open. 2025;8(9):e2533118. doi:10.1001/jamanetworkopen.2025.33118 40982278 PMC12455376

[31] Sule S, DaCosta MC, DeCou E, Gilson C, Wallace K, Goff SL. Communication of COVID-19 misinformation on social media by physicians in the US. JAMA Netw Open. 2023;6(8):e2328928. doi:10.1001/jamanetworkopen.2023.28928 37581886 PMC10427940

[32] Sundelson AE, Jamison AM, Huhn N, Pasquino SL, Sell TK. Fighting the infodemic: the 4 i Framework for Advancing Communication and Trust. BMC Public Health. 2023;23(1):1662. doi:10.1186/s12889-023-16612-9 37644563 PMC10466697

[33] van der Linden S. Misinformation: susceptibility, spread, and interventions to immunize the public. Nat Med. 2022;28(3):460-467. doi:10.1038/s41591-022-01713-6 35273402

[34] Kozyreva A, Lorenz-Spreen P, Herzog SM, . Toolbox of individual-level interventions against online misinformation. Nat Hum Behav. 2024;8(6):1044-1052. doi:10.1038/s41562-024-01881-0 38740990

[35] Palmer A, Lopez G, Taylor JS, Gallagher CM. Addressing misinformation about cancer care in the clinical setting: a pilot study of a conversation guide and training for cancer care providers. J Clin Oncol. 2024;42(16)(suppl):e23250-e23250. doi:10.1200/JCO.2024.42.16_suppl.e23250

[36] Lazard AJ, Queen TL, Pulido M, . Social media prompts to encourage intervening with cancer treatment misinformation. Soc Sci Med. 2025;372:117950. doi:10.1016/j.socscimed.2025.117950 40096813

[37] Okuhara T, Okada H, Yokota R, Kiuchi T. Effectiveness and determinants of narrative-based corrections for health misinformation: a systematic review. Patient Educ Couns. 2025;139:109253. doi:10.1016/j.pec.2025.109253 40651127

[38] Hyatt A, Shelly A, Cox R, Humphries E, Lock G, Varlow M. How can we improve information for people affected by cancer? A national survey exploring gaps in current information provision, and challenges with accessing cancer information online. Patient Educ Couns. 2022;105(8):2763-2770. doi:10.1016/j.pec.2022.04.009 35465976

[39] Perlis RH, Lunz Trujillo K, Green J, . Misinformation, trust, and use of ivermectin and hydroxychloroquine for COVID-19. JAMA Health Forum. 2023;4(9):e233257. doi:10.1001/jamahealthforum.2023.3257 37773507 PMC10542734

[40] Cervantes L, Martin M, Frank MG, . Experiences of Latinx individuals hospitalized for COVID-19: a qualitative study. JAMA Netw Open. 2021;4(3):e210684. doi:10.1001/jamanetworkopen.2021.0684 33704475 PMC7953277

[41] Leader AE, Burke-Garcia A, Afanaseva D, . Partnering with social media influencers to promote HPV vaccination in diverse communities. Vaccine. 2025;53:127085. doi:10.1016/j.vaccine.2025.127085 40186996

