# Supplementary material for: Fear and Medical Misinformation Regarding Risk of Progression or Recurrence Among Patients with Breast Cancer
Source: JAMA Netw Open. 2025 Dec 29;8(12):e2549809. doi: 10.1001/jamanetworkopen.2025.49809 (PMC12750251; doi:10.1001/jamanetworkopen.2025.49809)
Supplement: Supplement 1. — eTable. Multivariable Logistic Regression of Variables Associated with Awareness of Misinformation [file jamanetwopen-e2549809-s001.pdf]

Supplemental Online Content

Miller DG, Lapen K, Dee EC, et al. Fear and medical misinformation regarding risk of progression or recurrence among patients with breast cancer. *JAMA Netw Open.* 2025;8(12):e2549809. doi:10.1001/jamanetworkopen.2025.49809

eTable 1. Multivariable Logistic Regression of Variables Associated with Awareness of Misinformation

This supplemental material has been provided by the authors to give readers additional information about their work.

© 2025 Miller DG et al. *JAMA Network Open*.

**eTable 1.** Multivariable Logistic Regression of Variables Associated with Awareness of Misinformation		
**Variable**	**Adjusted OR (95% CI)**	**P-Value**
Age		
Per 10-year increase	0.83 (0.63-1.09)	0.19
Race & Ethnicity		
Non-Hispanic White	—	—
Asian	0.59 (0.20-2.01)	0.36
Hispanic/Latino	2.29 (0.76-9.92)	0.19
Non-Hispanic Black	1.21 (0.50-3.24)	0.69
Other/Unknown	0.59 (0.21-1.78)	0.32
Highest Level of Education		
Postgraduate Degree	—	—
Associate/Bachelors	0.79 (0.52-1.20)	0.27
Some College	1.13 (0.66-1.96)	0.67
High School	0.8 (0.40-2.08)	0.77
Marital Status		
Married/Partnered	—	—
Single	0.64 (0.34-1.23)	0.17
Divorced/Separated	0.85 (0.47-1.56)	0.59
Widowed	1.73 (0.70-4.94)	0.27
Breast Cancer Status		
Early-Stage	—	—
Locally Advanced	1.20 (0.80-1.81)	0.38
Metastatic	1.32 (0.64-2.89)	0.46
Treatment Status		
On Active Treatment	—	—
Treatment Naïve	1.02 (0.36-3.34)	0.98
Completed Treatment	1.18 (0.80-1.74)	0.41
Employment Status		

© 2025 Miller DG et al. *JAMA Network Open*.

Employed	—	—
Retired	0.94 (0.53-1.66)	0.83
Homemaker	0.98 (0.32-3.68)	0.97
Student	*[Too few participants]*	—
On Leave or Disability	1.11 (0.47-2.88)	0.82
Unemployed	1.78 (0.55-8.01)	0.38
Insurance Status		
Private Insurance	—	—
Medicare	1.06 (0.58-1.93)	0.85
Medicaid	1.09 (0.41-3.28)	0.87
No Insurance	*[Too few participants]*	—
Other	*[Too few participants]*	—
Treatment Adherence		
Adherent	—	—
Non-adherent	1.04 (0.68-1.62)	0.86
Clinically Meaningful Fear of Recurrence		
No	—	—
Yes	1.08 (0.73-1.60)	0.71

© 2025 Miller DG et al. *JAMA Network Open*.
