# Supplementary material for: Fear and Medical Misinformation Regarding Risk of Progression or Recurrence Among Patients with Breast Cancer
Source: JAMA Netw Open. 2025 Dec 29;8(12):e2549809. doi: 10.1001/jamanetworkopen.2025.49809 (PMC12750251; doi:10.1001/jamanetworkopen.2025.49809)
Supplement: Supplement 2. — Data Sharing Statement [file jamanetwopen-e2549809-s002.pdf]

Data Sharing Statement

Miller. Fear and Medical Misinformation Regarding Risk of Progression or Recurrence Among Patients with Breast Cancer. *JAMA Netw Open*. Published December 29, 2025.
doi:10.1001/jamanetworkopen.2025.49809

Data
Data available: Yes
Data types: Deidentified participant data
How to access data: Interested individuals may contact the corresponding author (David Miller, millerd4@mskcc.org) to request access to anonymized study data.
When available: With publication

Supporting Documents
Document types: None

Additional Information
Who can access the data: Researchers whose proposed use of the data has been approved
Types of analyses: For research-related purposes
Mechanisms of data availability: With investigator support and signed data access agreement.
